# Physiological Traits of Dihomo-γ-Linolenic Acid Production of the Engineered *Aspergillus oryzae* by Comparing Mathematical Models

**DOI:** 10.3389/fmicb.2020.546230

**Published:** 2020-11-05

**Authors:** Sompot Antimanon, Jutamas Anantayanon, Siwaporn Wannawilai, Bhimabol Khongto, Kobkul Laoteng

**Affiliations:** Industrial Bioprocess Technology Research Team, Functional Ingredients and Food Innovation Research Group, National Center for Genetic Engineering and Biotechnology (BIOTEC), National Science and Technology Development Agency (NSTDA), Khlong Luang, Thailand

**Keywords:** dihomo-γ-linolenic acid, *Aspergillus oryzae*, cassava starch hydrolysate, mother liquor, kinetic model, lipid accumulation

## Abstract

Dihomo-γ-linolenic acid (DGLA; C20:3 *n*-6) is expected to dominate the functional ingredients market for its role in anti-inflammation and anti-proliferation. The DGLA production by the engineered strain of *Aspergillus oryzae* with overexpressing *Pythium* Δ^6^-desaturase and Δ^6^-elongase genes was investigated by manipulating the nutrient and fermentation regimes. Of the nitrogen sources tested, the maximum biomass and DGLA titers were obtained in the cultures using NaNO_3_ grown at pH 6.0. For establishing economically feasible process of DGLA production, the cost-effective medium was developed by using cassava starch hydrolysate (CSH) and NaNO_3_ as carbon and nitrogen sources, respectively. The supplementation with 1% (*v*/*v*) mother liquor (ML) into the CSH medium promoted the specific yield of DGLA production (*Y_*DGLA*__/__*X*_*) comparable with the culture grown in the defined NaNO_3_ medium, and the DGLA proportion was over 22% in total fatty acid (TFA). Besides, the GLA was also generated at a similar proportion (about 25% in TFA). The mathematical models of the cultures grown in the defined NaNO_3_ and CSH/ML media were generated, describing that the lipid and DGLA were growth-associated metabolites corresponding to the relevant kinetic parameters of fermentations. The controlled mode of submerged fermentation of the engineered strain was explored for governing the PUFA biosynthesis and lipid-accumulating process in relation to the biomass production. This study provides an informative perspective in the *n-*6 fatty acid production through physiological manipulation, thus leading to a prospect in viable production of the DGLA-enriched oil by the engineered strain.

## Introduction

Increasing concern on health and well-being by global consumers has stimulated the holistic process development in production of functional ingredients for creation of specialty and healthier products. Functional lipids are of current subjects involved in a broad spectrum of metabolic conditions in human and animal systems. Long-chain polyunsaturated fatty acids (LC-PUFAs) in *n*-3 and *n*-6 series are attracting attention because of their physiological and structural roles in such biological systems. Of them, dihomo-γ-linolenic acid (DGLA; C20:3 *n*-6) is a direct fatty acid precursor for biosynthesis of biologically active eicosanoids (1-series prostaglandin and thromboxanes) and other metabolites, which exert clinical efficacies, such as anti-inflammatory (Nakamura et al., 1993), anti-thrombotic (Smith et al., 1989), anti-hypertensive (Hassall and Kirtland, 1984), and anti-allergic (Taki et al., 1993) activities (Tabolacci et al., 2010; Wang et al., 2012; Sergeant et al., 2016). Moreover, there are scientific reports on a potential use of this *n*-6 PUFA in anti-cancer activity as its role in cell apoptosis and anti-proliferation (Wang et al., 2012; Xu and Qian, 2014). Interestingly, the safety of DGLA intake without adverse effect has been documented (Umeda-Sawada et al., 2006). Indeed, the process development of DGLA production is required to meet the projected growing demands. Because of the fact that the 20-carbon PUFA is not generally found in common foods, it is particularly important to overcome such constraint through biotechnological production using emerging tools and technologies.

The *n*-3 and *n*-6 LC-PUFA biosynthetic pathways consist of a series of metabolic reactions catalyzed by membrane-bound desaturase and elongase enzymes, which are specific to the length and existing double bond position in fatty acyl chains. For the main route of *n*–6 PUFA biosynthesis, linoleic acid (C18:2 *n*-6; LA) is a basic substrate for biotransforming to γ-linolenic acid (C18:3 *n*-6; GLA) and DGLA by Δ^6^-desaturase and Δ^6^-elongase, respectively. DGLA is then used as an intermediate for further desaturation and elongation, yielding highly unsaturated fatty acids. This metabolic flux is a cause of low accumulation of DGLA in the cells. The biotechnological production by oleaginous strains as cell factories has been proven to be a potential strategy for commercial production of other LC-PUFAs, such as arachidonic acid (ARA; C20:4 *n*-6) and docasahexaenoic acid (DHA; C22:6 *n*-3). It has been reported that the maximum PUFA contents are usually obtained when growing the microbial cells at lipid-accumulating phase, which is a nutrient imbalance stage by carbon-excessive and nitrogen-limited conditions (Sunja et al., 2011; Masurkar and Vakil, 2015; Singhasuwan et al., 2015). Several abiotic factors also affect PUFA production, such as pH, dissolved oxygen, and culture temperature (Laoteng et al., 2011). Recently, the oleaginous fungus, *Aspergillus oryzae*, was selected as a cell chassis for DGLA production as its phenotypic and genotypic features in oleaginicity (Vorapreeda et al., 2012; Chutrakul et al., 2016). The strain improvement was implemented by co-expressing the *Pythium* Δ^6^-desaturase and Δ^6^-elongase genes under the control of *gpdA* and *toxA* promoters, respectively (Chutrakul et al., 2016). Nevertheless, the nutritional and environmental controls for improving the production of DGLA-containing oils are required in addition to the genetic modification. Moreover, the techno-economic feasibility in the production process should be taken into consideration to realistic application. One of the cost factors in the microbial production process is culture medium that can be rationally designed to encounter the commercial production. As such, agro-industrial residues are of particular interest in terms of cost competitiveness and renewable feedstock for microbial production. Besides, the fermentation conditions should be established by using the selected feedstock as either carbon or nitrogen sources to ensure the capability in fungal growth and LC-PUFA production. With description of the metabolic behavior of microbial cells, the mathematical modeling is also a beneficial tool for prediction of fermentation process by fine-tuning the kinetic parameters to address particular purposes (Yang et al., 2011; Marudkla et al., 2018).

This work aimed to investigate the physiological control of the engineered *A. oryzae* strain with overexpressing the *Pythium* Δ^6^-desaturase and Δ^6^-elongase genes for DGLA production. Using cassava starch hydrolysate (CSH) and the by-product derived from monosodium glutamate production process as cheap nutrient sources, the controlled mode of submerged fermentation by the engineered fungal strain was explored for governing PUFA biosynthesis and lipid-accumulating process along with optimizing the mycelial growth. Further, performances of biomass and DGLA production in the engineered strain of *A. oryzae* at optimal conditions were explained through the constructed mathematical models. This work provides an informative perspective in fungal fermentation of LC-PUFA by the metabolically engineered strain that would be applicable for larger-scale production.

## Materials and Methods

### Strain and Cultivation

The DGLA-producing recombinant strain of *A. oryzae*, which was previously generated (Chutrakul et al., 2016) by overexpressing the codon-optimized Δ^6^-desaturase and Δ^6^-elongase genes of *Pythium*, was used in this study. The fungal strain was routinely cultivated on PDA agar (BD, United States) at 30°C for 5–7 days.

### Inoculum Preparation

The fungal culture grown on streamed rice grains at 30°C for 5–7 days was subjected to inoculum preparation. The spores were harvested by adding 0.05% (*v*/*v*) Tween 80 to the culture and then filtered through Miracloth (Merck, Germany). The spore suspension was inoculated into broth medium at the final concentration of 10^6^ spores/ml.

### Submerged Fermentation of the Engineered Strain of *A*. *oryzae*

A basal medium with initial pH of 5.0 was prepared accordingly, in which 1 L consisted of 60.0 g glucose, 2.4 g KH_2_PO_4_, 0.5 g MgSO_4_⋅7H_2_O, 0.1 g CaCl_2_⋅7H_2_O, 10 mg MnSO_4_⋅H_2_O, 0.5 mg CuSO_4_⋅5H_2_O, 15 mg FeCl_3_⋅6H_2_O, and 7.5 mg ZnSO_4_⋅7H_2_O. For varying the nitrogen sources, organic nitrogen (yeast extract), inorganic ammonia sources [NH_4_Cl, (NH_4_)_2_SO_4_, and (NH_4_)_2_HPO_4_] and inorganic nitrate sources (KNO_3_ and NaNO_3_) were added into the basal medium to obtain a C/N ratio of 30. The fungal cultures were carried out in a 250-ml Erlenmeyer flask containing 50 ml broth medium and grown at 30°C with shaking at 250 rpm.

To investigate the effect of culture pH on DGLA production, the fungal fermentation was performed in a 5-L bioreactor (Biostat B-DCU; Sartorius Stedim Biotech, Germany) containing 2 L of the defined NaNO_3_ medium (basal medium with NaNO_3_). The pH values of individual cultures were constantly controlled at 3, 4, 5, 6, and 7 during the cultivation by using either 2 M HCl or 2 M KOH solutions. The fermentation condition was controlled at 30°C, 1 vvm of aeration rate, and 300–500 rpm of agitation rate.

### Fungal Cultivation With Cheap Medium

Using the method modified from Wei et al. (2009), CSH was prepared by hydrolyzing the cassava starch using enzymatic reactions of α-amylase (4.0 × 10^4^ U/ml) and glucoamylase (1.5 × 10^5^ U/ml). The enzymes were purchased from iKnowZyme company (Thailand). The CSH medium with final concentration of 6% (*v*/*v*) glucose was prepared by adding the prepared CSH (30%, *v*/*v*) into the defined NaNO_3_ medium. The by-product obtained from the production process of monosodium glutamate, called mother liquor (ML), was chosen as a nutrient supplement for fungal cultivation. Four levels of ML, 0.5, 1.0, 3.0, and 5.0% (*v*/*v*), were added into the CSH medium.

### Analyses of Biomass, Fatty Acid Composition, Lipid Titer, and Residual Glucose Concentration

Biomass concentration or dry cell weight (DCW) was measured by filtering the mycelial culture through a filter paper (Whatman No. 1). The mycelial cells were washed with distilled water and then dried at 60°C in a hot-air oven to obtain a constant weight.

Dried mycelia were ground and then directly subjected to the preparation of fatty acid methyl ester (FAME) using the direct transmethylation method modified from Lepage and Roy (1984). The FAMEs were analyzed by gas chromatography (GC-7890B; Agilent Technologies, United States) equipped with a flame ionization detector. Using the HP-88 capillary column (100 m × 250 μm × 0.2 μm; Agilent Technologies), the column temperature was programmed from 140 to 240°C with an increasing rate of 4°C/min. The detector temperature was set at 240°C, and the flow rate of carrier gas (helium) was controlled at 1.0 ml/min. Pentadecaenoic acid (C15:0) was used as an internal standard. The amount of individual fatty acids was quantified by calculating the respective chromatographic area.

Total lipid extraction was performed using a mixture solution of chloroform and methanol (2:1, *v*/*v*) (Folch et al., 1957). The sample was shaken for 90 min and then centrifuged for 10 min. After solvent evaporation under vacuum in rotary evaporator, lipid concentration was determined by weighting.

Residual glucose concentration in the culture broth was measured by high performance liquid chromatography (UHPLC Ultimate 3000; Thermo Fisher Scientific) equipped with a refractive index detector (Refractomax 520; Dataapex) and Aminex HPX-87H column (300 × 7.8 mm; Aminex). The column temperature was controlled at 60°C. After filtering through a 0.2-μm cellulose acetate membrane, the sample (10 μl) was subjected to the injection. Sulfuric acid solution (18 mM) was used as a mobile phase with a flow rate of 0.6 ml/min.

### Statistical Analysis of Experimental Data

All experiments were performed in triplicates independently, and the data were expressed as means ± SD. Statistical Program for Social Sciences (SPPS) software version 11.5 (SPSS software products, United States) was used for statistical analysis of data. The data were considered statistically significant at *P* < 0.05.

### Mathematical Modeling of the Fungal Growth and DGLA Production

For describing growth behavior of the engineered strain of *A. oryzae*, the biomass concentrations obtained from various cultivation conditions were subjected to generate mathematical models by logistic equations (Yang et al., 2011; Marudkla et al., 2018), as follows:

(1) $$\frac{d\,X}{d\,t}=\mu_{\max}\,X\,\left(1-\frac{X}{X_{m}}\right)$$

where $\frac{d\,X}{d\,t}$ is the rate of fungal growth (g_*X*_/L.h), μ_*max*_ is the maximum specific growth rate, *X* is the biomass concentration, and *X*_*m*_ is the maximum biomass concentration. In Eq. (1), *μ_*max*_* and *X*_*m*_ are constant values of the logistic model.

The substrate consumption kinetic ($\frac{d\,S}{d\,t}$) was taken from the fungal growth rate and cell maintenance (g_*S*_/L.h). Therefore, the substrate consumption rate was modeled as follows:

(2) $$\frac{d\,S}{d\,t}=-\left[\left(\frac{1}{Y_{X/S}}\right)\,\left(\frac{d\,X}{d\,t}\right)+m_{S}\,X\right]$$

where *Y_*X*__/__*S*_* is a biomass yield on substrate and *m*_*S*_ is the biomass maintenance coefficient.

The production rates of DGLA and lipid were described by Luedeking–Piret equation (Luedeking and Piret, 1959), which depended on biomass concentration and specific growth rate (*μ*). With respect to the experimental data, the DGLA and lipid production rate decreased when substrate was exhausted as follows:

(3) $$\frac{d\,P}{\mathrm{dt}}=\left(\alpha \,\mu +\beta \right)\,X-k_{d}\,{\left(1-\frac{S}{S_{0}}\right)}^{n}\,X$$

where $\frac{d\,\text{P}}{d\,t}$ is the DGLA or lipid production rate (g_*D*_/L.h and g_*L*_/L.h, respectively); α and β are the growth-associated and non-growth associated product formation coefficients, respectively. The formation mode of each product was defined according to the fungal growth. If α≠ 0 and β = 0, the product formation is the growth-related production. On the contrary, if α **=** 0 and β≠ 0, the product formation is unrelated to fungal growth. In case if the product formation is mixed with the growth production, the value α≠ 0 and β≠ 0. *k*_*d*_ is specific product turnover rate, and *n* is degree of product depletion.

### Estimation of Model Parameters and Statistical Analyses

The experimental data were used to estimate the fermentation kinetic parameters (i.e., *μ_*max*_*, *Y_*X*__/__*S*_*, *m*_*S*_, α, and β) of the aforementioned mathematical models. The fitting between the model equations and experimental data was performed using Berkeley Madonna software^1^. The value of determination coefficient (*R*^2^) was set to assign the best fit between the model and the experimental data (Wannawilai et al., 2017). The *R*^2^ was calculated using the following equation:

(4) $$R^{2}=\frac{\sum {\left(C_{\mathrm{cal}}-{\bar{C}}_{\exp}\right)}^{2}}{\sum {\left(C_{\mathrm{cal}}-{\bar{C}}_{\exp}\right)}^{2}+\sum {\left(C_{\mathrm{cal}}-{\bar{C}}_{\exp}\right)}^{2}}$$

In Eq. (4), *C*_*cal*_ is the value with a given variable calculated from the model, and *C*_*exp*_ is the corresponding experimentally measured value. ${\bar{C}}_{e\,x\,p}$ is an average of a set of experimentally measured values under a given variable.

## Results

### DGLA Production Yield of the Engineered *A*. *oryzae* Strain Depending on Nitrogen Sources

Using various inorganic nitrogen sources, it was found that there was a significant difference in maximum biomass concentrations (*p* < 0.05) of the engineered *A. oryzae* strain harboring *Pythium* Δ^6^-desaturase and Δ^6^-elongase genes, which are consistent with respective kinetic parameters, including *μ_*max*_*, *Y_*X*__/__*S*_*, and volumetric rate of biomass productivity (*Q*_*X*_). As shown in Table 1, the basal media containing ammonium sources [NH_4_Cl, (NH_4_)_2_HPO_4_, and (NH_4_)_2_SO_4_] were not favorable for the biomass production as compared with the nitrate-containing media (KNO_3_ and NaNO_3_). The growths of the fungal cultures using inorganic nitrate sources were markedly enhanced for about 2-fold increase of biomass concentration as compared with those of ammonium-containing cultures. Of the nitrogen sources tested, the highest biomass titer (23.90 g/L) was observed in the fungal culture using sodium nitrate (C/N = 30) for 48 h, which was the glucose-exhausted stage, indicating that this fungal cultivation was a carbon-limited condition. Unexpectedly, the fungal biomass concentration of the engineered strain using yeast extract was not comparable with those using nitrate sources.

**TABLE 1:** Fermentation parameters and DGLA production of the shake-flask cultures of the engineered *A. oryzae* using different nitrogen sources.

Parameters	Nitrogen source					
	Yeast extract	KNO_3_	NaNO_3_	(NH_4_)_2_HPO_4_	NH_4_Cl	(NH_4_)_2_SO_4_
*X*_*m*_ (g/L)	16.87 ± 1.05^*c*^	23.90 ± 1.10^*b*^	24.75 ± 1.32^*a*^	11.92 ± 1.20^*e*^	12.86 ± 1.11^*d*^	13.18 ± 0.64^*d*^
μ_*max*_ (day^–1^)	0.28 ± 0.01^*c*^	0.36 ± 0.03^*b*^	0.43 ± 0.02^*a*^	0.18 ± 0.00^*e*^	0.23 ± 0.02^*d*^	0.29 ± 0.01^*d*^
*Y*_*X/S*_ (g*_*X*_*/g*_*S*_*)	0.28 ± 0.01^*c*^	0.39 ± 0.02^*b*^	0.41 ± 0.02^*a*^	0.19 ± 0.00^*e*^	0.21 ± 0.01^*de*^	0.22 ± 0.02^*d*^
*Qx* (g*_*X*_*/L.h)	0.31 ± 0.02^*b*^	0.50 ± 0.03^*a*^	0.52 ± 0.02^*a*^	0.22 ± 0.03^*b*^	0.24 ± 0.01^*b*^	0.24 ± 0.01^*b*^
DGLA titer (mg/L)	92.78 ± 1.24^*c*^	100.38 ± 1.32^*b*^	116.32 ± 2.08^*a*^	35.35 ± 2.05^*f*^	45.42 ± 1.17^*e*^	55.35 ± 2.67^*d*^
GLA titer (mg/L)	230.91 ± 2.75^*c*^	263.90 ± 2.83^*b*^	284.62 ± 3.88^*a*^	101.83 ± 0.38^*e*^	127.70 ± 1.69^*d*^	139.70 ± 1.39^*d*^
Lipid titer (mg/L)	2946.27 ± 15.32^*b*^	2820.44 ± 34.32^*b*^	3193.01 ± 37.89^*a*^	495.36 ± 13.88^*c*^	294.32 ± 11.88^*d*^	396.78 ± 3.51^*cd*^
DGLA content (% in DCW)	0.55 ± 0.09^*a*^	0.42 ± 0.04^*c*^	0.47 ± 0.05^*b*^	0.29 ± 0.02^*e*^	0.35 ± 0.02^*d*^	0.42 ± 0.03^*c*^
GLA content (% in DCW)	1.36 ± 0.11^*a*^	1.10 ± 0.01^*bc*^	1.15 ± 0.01^*b*^	0.85 ± 0.03^*e*^	0.99 ± 0.03^*d*^	1.06 ± 0.02^*cd*^
Lipid content (% in DCW)	19.60 ± 0.00^*a*^	11.80 ± 0.00^*b*^	12.90 ± 0.14^*b*^	4.80 ± 0.00^*c*^	2.60 ± 0.57^*d*^	3.40 ± 0.28^*cd*^

**All data are presented as mean values with SD. Values marked with different superscript letters in the same row are significantly different (p < 0.05).**

Considering the LC-PUFA production in the engineered *A. oryzae* strain, we counted both DGLA and GLA as targeted products because the generated GLA product could be subsequently converted to DGLA by the expressed *Pythium* Δ^6^-elongase activity. Moreover, GLA product derived from the catalysis of *Pythium* Δ^6^-desaturase is also a potent *n*-6 PUFA with health benefit claims (Alabdulkarim et al., 2012; Xu and Qian, 2014; Sergeant et al., 2016). Similar to the biomass production, the highest DGLA and GLA titers were observed in the culture using NaNO_3_ as a nitrogen source (Table 1). Undoubtedly, the cultivations with ammonium sources exhibited low titers of DGLA and GLA that were a result of the poor growths as aforementioned. Although all cultures had a similar fatty acid profile, the DGLA proportion in total fatty acid (% in TFA) of the nitrate- and yeast extract–containing cultures were slightly higher than that of the cultures using ammonium sources (Supplementary Table S1). In addition, the lipid contents in dry cell weight (% in DCW) of the ammonium-containing cultures were lower than those using nitrate sources, which coincided with the lipid titers (Table 1). Among the nitrate sources, the fungal culture using NaNO_3_ also provided the highest lipid titer.

### DGLA Production of the Engineered*A*. *oryzae* Strain Related to Its pH-Associated Growth

**FIGURE 1:**
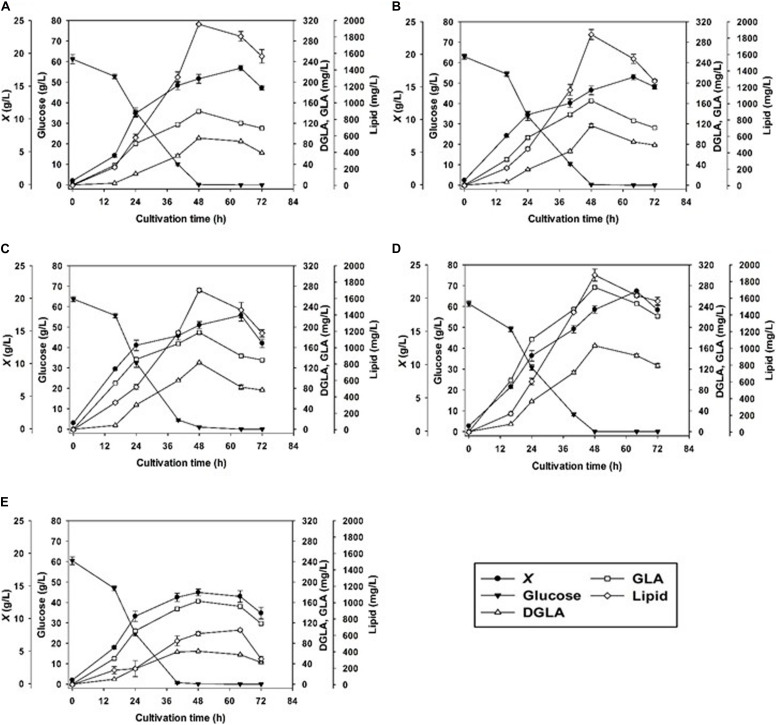
Cell growth DGLA, and GLA production of the engineered strain of *A. oryzae* by batch cultivation controlling different pH values, pH 3 **(A)**, 4 **(B)**, 5 **(C)**, 6 **(D)**, and 7 **(E)**.

In addition to the nitrogen sources, we investigated how the pH condition affect the mycelia growth and DGLA production. By controlling the constant pH value during the fermentation in a stirred-tank bioreactor, the results showed that the fungal growths rapidly increased at the first 24 h, which were observed in all NaNO_3_-containing cultures grown at different pH conditions. The maximum biomass concentrations (16.5–21.0 g/L) were observed in the 64-h cultures except for the culture grown at pH 7.0 (Figure 1). As a result, pH 6.0 was optimal for biomass production of the engineered strain. Notably, the biomass titer was rather low (13.4 ± 0.9 g/L) when cultivated at the neutral pH condition (pH 7.0) even though glucose was completely consumed.

The DGLA and GLA productions were significantly attributed by pH control. With the exception of the culture grown at pH 7.0, the titers of these *n*-6 fatty acids increased with the increase of pH values (pH 3.0–6.0). The highest titers of DGLA and GLA, 164.8 ± 1.8 and 276.9 ± 2.4 mg/L, respectively, were achieved by cultivating at the constant pH 6.0 condition for 48 h (Table 2). The lipid contents in all cultures were highest when glucose was exhausted (48-h cultivation). At pH 6.0, it was observed that the DGLA and GLA proportions in TFA were highest as compared with the cultures grown at other pH conditions (Table 3). In addition, the highest lipid titer (1877.43 ± 91.73 mg/L) was obtained by constantly controlling pH at 6.0, in which the specific yields of DGLA (*Y_*DGLA*__/__*X*_*) and GLA (*Y_*GLA*__/__*X*_*) were 7.8 ± 0.0, and 13.2 ± 0.3 mg/g DCW, respectively.

**TABLE 2:** Fermentation parameters and DGLA production of the engineered *A. oryzae* cultures grown in a stirred-tank bioreactor controlling constant pH condition at different values (the NaNO_3_-defined medium was used for fungal cultivation).

Parameters	Controlled pH value				
	3.0	4.0	5.0	6.0	7.0
*X*_*m*_ (g/L)	17.73 ± 0.40^*ab*^	16.20 ± 0.40^*b*^	17.40 ± 0.89^*b*^	21.03 ± 0.10^*a*^	13.38 ± 0.89^*c*^
μ_*max*_ (day^–1^)	0.12 ± 0.01^*a*^	0.12 ± 0.01^*a*^	0.12 ± 0.02^*a*^	0.13 ± 0.00^*a*^	0.11 ± 0.00^*a*^
*Y*_*X/S*_ (g*_*X*_*/g*_*S*_*)	0.29 ± 0.01^*ab*^	0.27 ± 0.01^*bc*^	0.29 ± 0.01^*ab*^	0.35 ± 0.02^*a*^	0.22 ± 0.00^*c*^
*Qx* (g*_*X*_*/L.h)	0.34 ± 0.01^*ab*^	0.31 ± 0.01^*ab*^	0.33 ± 0.02^*ab*^	0.38 ± 0.01^*a*^	0.29 ± 0.02^*b*^
DGLA titer (mg/L)	91.08 ± 0.36^*d*^	116.43 ± 3.53^*c*^	130.32 ± 1.43^*b*^	164.83 ± 1.80^*a*^	64.57 ± 0.47^*e*^
GLA titer (mg/L)	143.30 ± 0.30^*d*^	165.30 ± 1.20^*c*^	189.30 ± 1.20^*b*^	276.86 ± 2.40^*a*^	162.61 ± 2.10^*c*^
Lipid titer (mg/L)	2003.87 ± 37.00^*a*^	1843.23 ± 61.38^*a*^	1700.83 ± 20.32^*a*^	1877.43 ± 91.73^*a*^	1290.80 ± 34.65^*b*^
*Y*_*DGLA/X*_ (mg/g*_*X*_*)	5.14 ± 0.21^*c*^	7.18 ± 0.11^*b*^	7.48 ± 0.10^*b*^	7.83 ± 0.03^*a*^	4.82 ± 0.22^*c*^
*Y*_*GLA/X*_ (mg/g*_*X*_*)	8.08 ± 0.13^*c*^	10.20 ± 0.14^*b*^	10.87 ± 0.21^*b*^	13.16 ± 0.25^*a*^	12.15 ± 0.31^*a*^
DGLA content (% in DCW)	0.57 ± 0.12^*c*^	0.80 ± 0.12^*b*^	0.82 ± 0.11^*b*^	0.90 ± 0.02^*a*^	0.46 ± 0.12^*c*^
GLA content (% in DCW)	0.89 ± 0.11^*bc*^	1.14 ± 0.09^*b*^	1.19 ± 0.10^*b*^	1.52 ± 0.13^*a*^	1.16 ± 0.12^*b*^
Lipid content (% in DCW)	12.44 ± 1.33^*a*^	12.74 ± 1.10^*a*^	10.70 ± 0.88^*a*^	10.28 ± 1.14^*a*^	9.22 ± 0.11^*a*^

**All data are presented as mean values with SD. Values marked with different superscript letters in the same row are significantly different (p < 0.05).**

**TABLE 3:** Fatty acid composition of the engineered *A. oryzae* cultures grown in a stirred-tank bioreactor by controlling different pH conditions (the NaNO_3_-defined medium was used for fungal cultivation).

pH	Fatty acid composition in total fatty acid (%, *w*/*w*)							
	C16:0	C18:0	C18:1 *n*-9	C18:2 *n*-6	C18:3 *n*-6	C20:3 *n*-6	Others	Total PUFA
3	20.1 ± 0.1	10.6 ± 0.1	26.7 ± 0.2	20.9 ± 0.3	15.6 ± 0.2	6.2 ± 0.1	0.0 ± 0.0	42.7 ± 0.6
4	18.8 ± 2.2	9.7 ± 0.2	26.6 ± 1.2	17.3 ± 1.0	16.8 ± 0.4	8.9 ± 0.2	2.0 ± 0.1	43.0 ± 1.2
5	16.8 ± 1.8	9.3 ± 0.2	22.4 ± 1.1	22.8 ± 1.3	18.8 ± 0.3	8.2 ± 0.3	1.8 ± 0.1	49.7 ± 1.8
6	16.0 ± 1.0	8.5 ± 0.4	20.3 ± 0.4	25.5 ± 1.2	19.2 ± 0.4	10.6 ± 0.2	0.0 ± 0.0	55.3 ± 2.0
7	23.5 ± 0.3	10.0 ± 0.4	20.6 ± 0.3	19.9 ± 0.8	17.4 ± 0.4	6.8 ± 1.1	1.2 ± 0.1	44.1 ± 1.1

**All data are presented as mean values with SD.**

### Enhancing the DGLA Production by Using the Cost-Effective Medium

The cultivation of the engineered *A. oryzae* using glucose as a carbon source seems to be a promising process for DGLA production. However, glucose is still rather expensive, which might not be economically feasible for the 20C-PUFA production. As such, the NaNO_3_-defined medium was modified by replacing the glucose powder with an inexpensive carbon substrate (CSH). As shown in Table 4, the CSH medium (0% ML) could be used for the fungal growth; however, the biomass and DGLA productions were lower than the NaNO_3_-defined medium. Interestingly, about 1.6-fold increase of DGLA proportion (16.1% DGLA in TFA) was found in the CSH-grown culture when compared with the culture using NaNO_3_-defined medium. Conversely, the oleic acid (C18:1 *n*-9), which was a predominant fatty acid in the fungal cell, was proportionally decreased when using the CSH medium for cultivation. Total PUFA proportion of the culture using CSH medium was over 60% of TFA (Table 5), which was strikingly higher than that of the culture grown in NaNO_3_-defined medium (Table 3).

**TABLE 4:** Fermentation parameters and DGLA production of the engineered *A. oryzae* cultures grown in a stirred-tank bioreactor using the CSH medium (0% ML) and CSH medium supplemented with different ML amounts (the culture pH was constantly controlled at 6.0).

Parameters	ML amount (% *v*/*v*)				
	0.0	0.5	1.0	3.0	5.0
*X*_*m*_ (g/L)	13.92 ± 1.04^*c*^	15.41 ± 1.49^*bc*^	16.67 ± 1.30^*b*^	19.41 ± 0.78^*a*^	20.23 ± 0.45^*a*^
μ_*max*_ (day^–1^)	0.19 ± 0.03^*c*^	0.21 ± 0.02^*bc*^	0.22 ± 0.02^*abc*^	0.26 ± 0.01^*ab*^	0.27 ± 0.01^*a*^
*Y*_*X/S*_ (g*_*X*_*/g*_*S*_*)	0.23 ± 0.01^*c*^	0.25 ± 0.01^*bc*^	0.28 ± 0.02^*abc*^	0.32 ± 0.01^*ab*^	0.33 ± 0.01^*a*^
*Q*_*X*_ (g*_*X*_*/L.h)	0.19 ± 0.01^*b*^	0.23 ± 0.02^*ab*^	0.26 ± 0.01^*ab*^	0.30 ± 0.02^*a*^	0.31 ± 0.02^*a*^
DGLA titer (mg/L)	48.61 ± 2.55^*d*^	123.30 ± 2.48^*b*^	170.43 ± 1.55^*a*^	121.24 ± 1.21^*b*^	111.75 ± 1.86^*c*^
GLA titer (mg/L)	52.83 ± 2.58^*e*^	117.10 ± 2.28^*d*^	217.34 ± 4.21^*a*^	184.50 ± 2.60^*b*^	178.10 ± 5.33^*c*^
Lipid titer (mg/L)	626.40 ± 33.22^*c*^	838.38 ± 19.44^*b*^	1085.36 ± 20.44^*a*^	955.55 ± 16.55^*ab*^	920.30 ± 50.06^*b*^
*Y*_*DGLA/X*_ (mg/g*_*X*_*)	4.45 ± 0.21^*d*^	8.00 ± 0.33^*b*^	10.23 ± 0.65^*a*^	6.24 ± 0.11^*c*^	5.52 ± 0.05^*cd*^
*Y*_*GLA/X*_ (mg/g*_*X*_*)	4.83 ± 0.17^*d*^	7.60 ± 1.02^*c*^	13.03 ± 1.10^*a*^	9.40 ± 0.65^*b*^	8.80 ± 0.38^*b*^
DGLA content (% in DCW)	0.44 ± 0.01^*d*^	0.80 ± 0.02^*b*^	1.02 ± 0.02^*a*^	0.62 ± 0.01^*c*^	0.55 ± 0.02^*c*^
GLA content (% in DCW)	0.48 ± 0.02^*d*^	0.76 ± 0.02^*c*^	1.30 ± 0.05^*a*^	0.94 ± 0.03^*b*^	0.88 ± 0.03^*b*^
Lipid content (% in DCW)	4.50 ± 0.12^*c*^	6.35 ± 0.15^*a*^	6.51 ± 0.34^*a*^	4.92 ± 0.14^*b*^	4.55 ± 0.05^*b*^

**All data are presented as mean values with SD. Values marked with different superscript letters in the same row are significantly different (p < 0.05).**

**TABLE 5:** Fatty acid composition of the engineered *A. oryzae* cultures using the CSH medium (0% ML) and CSH medium supplemented with different ML amounts (the culture pH was constantly controlled at 6.0).

ML amount (% *v*/*v*)	Fatty acid composition (% in TFA)							
	C16:0	C18:0	C18:1 *n*-9	C18:2 *n*-6	C18:3 *n*-6	C20:3 *n*-6	Others	Total PUFA
0.0	20.5 ± 1.1	5.8 ± 0.2	11.2 ± 1.5	30.9 ± 2.3	14.4 ± 0.2	16.1 ± 0.5	1.2 ± 0.0	61.3 ± 1.0
0.5	20.0 ± 2.2	4.4 ± 0.8	5.7 ± 1.1	22.9 ± 1.8	25.0 ± 1.4	20.7 ± 2.2	1.3 ± 0.1	68.6 ± 1.3
1.0	20.0 ± 1.5	5.1 ± 0.3	6.7 ± 1.4	19.2 ± 2.5	25.4 ± 1.3	22.1 ± 2.3	1.4 ± 0.2	66.7 ± 1.1
3.0	19.9 ± 1.9	4.3 ± 0.1	7.5 ± 0.4	22.7 ± 1.3	26.7 ± 2.5	17.7 ± 1.3	1.2 ± 0.0	67.1 ± 1.0
5.0	21.1 ± 2.3	4.4 ± 0.3	7.1 ± 1.0	22.5 ± 1.9	27.3 ± 2.2	17.7 ± 1.2	0.0 ± 0.0	67.4 ± 1.2

**All data are presented as mean values with SD.**

To enhance the DGLA production yield, the agro-industrial residue (ML) enriched in some amino acids and other nutrients (Supplementary Table 2), which was a by-product derived from the monosodium glutamate production, was exploited as a nutrient supplement for the fungal cultivation. It was found that the ML supplementation significantly promoted the biomass production as clearly indicated by the kinetic growth parameters. The biomass titer increased with the increase of ML contents, and the highest biomass production (20.2 ± 0.5 g/L) was found in the culture supplemented with 5% (*v*/*v*) of ML, which was comparable with the cultures using NaNO_3_-defined medium. However, the highest titers of DGLA (170.4 ± 1.6 mg/L) and GLA (217.3 ± 4.2 mg/L) were observed in the culture grown in the CSH medium containing 1% ML (CSH/ML medium), which was also the same condition that the maximum lipid titer was obtained. Surprisingly, very high proportions of DGLA and GLA, 22.1 and 25.4% of TFA, respectively, were also found in the culture grown in the CSH/ML medium as shown in Table 5. Indeed, the maximum specific yield of DGLA production of the CSH/ML-grown culture was about 30.7% higher than the culture using NaNO_3_-defined medium.

### Comparative Kinetic Modeling of Biomass, Lipid, and DGLA Production Using Different Media

The kinetic models describing the growth and DGLA product formation were constructed for predicting and controlling the production process by the engineered strain of *A. oryzae*. The parameters related to biomass or cell growth (*X*_*m*_, μ_*max*_, *m*_*S*_, and *Y*_*X/S*_), DGLA production (α*_*D*_*, β*_*D*_*, *k*_*dD*_, and *n*_*D*_), lipid production (α*_*L*_*, β*_*L*_*, *k*_*dL*_, and *n*_*L*_), and residual glucose concentration (*S*) were measured and calculated at different time points during fungal fermentation. The data were then subjected to simulate the model according to the proposed mathematical equations (see “Materials and Methods” section; Eqs. 1–3).

**FIGURE 2:**
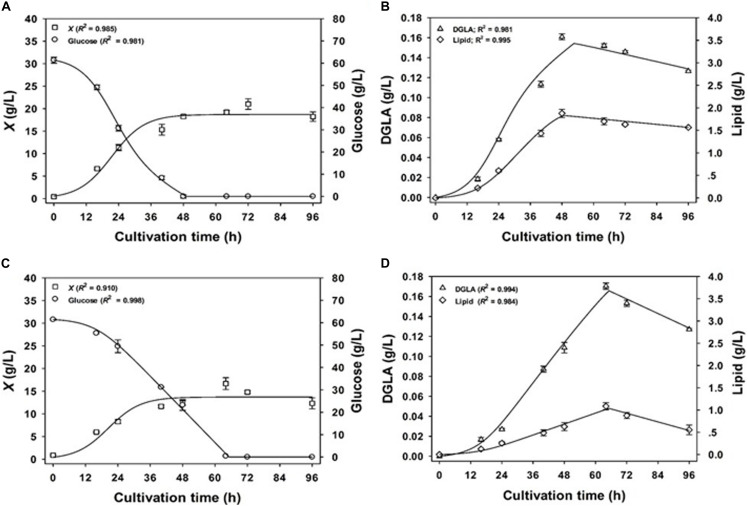
Comparison of the experimental data (symbols) and the calculated values (lines) of biomass concentration and DGLA production of the engineered *A. oryzae* cultures grown in NaNO_3_-defined medium **(A,B)** and CSH/ML medium **(C,D)**.

Figure 2 shows the fermentation model using NaNO_3_-defined medium. It was found that the estimated kinetic parameters and the measured values of experimental set were aligned well, indicating that the models fitted to the experimental data with the correlation coefficient (*R*^2^) values >0.98. All values of the best-fit model parameters are shown in Table 6. Considering the mycelial growth, glucose consumption, and lipid and DGLA production, the measured data indicated a linear relationship between the formation of products (DGLA and lipid) and the mycelia growth (when glucose was still available), in accordance with Eq. 3. Moreover, it was found that the mode of DGLA and lipid formation was growth associated as indicated by the β*_*D*_* and β*_*L*_* values close to zero.

**TABLE 6:** Kinetic parameters estimated by fitting the models to the experimental data of the engineered *A. oryzae* cultures grown in the NaNO_3_-defined and CSH/ML media.

Kinetic models		Parameters	Values	
			NaNO_3_-defined medium	CSH/ML medium
Cell growth (g_*X*_/L.h)	$\frac{d\,X}{d\,t}=\mu_{m\,a\,x}\,X\,\left(1-\frac{X}{X_{m}}\right)$	*μ_*max*_* (h^–1^)	0.16	0.17
		*X*_*m*_ (g/L)	18.39	13.72
Glucose consumption (g_*S*_/L.h)	$\frac{d\,S}{d\,t}=-\left[\left(\frac{1}{Y_{X/S}}\right)\,\left(\frac{d\,X}{d\,t}\right)+m_{S}\,X\right]$	*m*_*S*_ (g/g.h)	0.02	0.09
		*Y*_*X/S*_ (g*_*X*_*/g*_*S*_*)	0.34	0.21
DGLA production (g_*D*_/L.h)	$\frac{d\,P_{D}}{d\,t}=\left(\alpha_{D}\,\mu +\beta_{D}\right)\,X-k_{d\,D}\,{\left(1-\frac{S}{S_{0}}\right)}^{n_{D}}\,X$	α*_*D*_* (g/g)	5.26 × 10^–2^	1.51 × 10^–2^
		β*_*D*_* (g/g.h)	1.06 × 10^–8^	2.59 × 10^–8^
		*k*_*dD*_ (g/g.h)	1.38 × 10^–4^	3.48 × 10^–4^
		*n*_*D*_	1.32 × 10^–7^	6.20 × 10^–2^
Lipid production (g_*L*_/L.h)	$\frac{d\,P_{L}}{d\,t}=\left(\alpha_{L}\,\mu +\beta_{L}\right)\,X-k_{d\,L}\,{\left(1-\frac{S}{S_{0}}\right)}^{n_{L}}\,X$	α*_*L*_* (g/g)	3.94 × 10^–3^	9.70 × 10^–2^
		β*_*L*_* (g/g.h)	6.06 × 10^–8^	1.49 × 10^–8^
		*k*_*dL*_ (g/g.h)	6.38 × 10^–3^	2.66 × 10^–3^
		*n*_*L*_	3.33 × 10^–1^	1.17 × 10^–7^

However, the model parameters in NaNO_3_-defined medium could be not used for explaining the phenotypes found in the culture using CSH/ML medium because the correlation coefficient value was less than 0.65 (data not shown). This was a result of slower rates of glucose consumption, mycelia growth, and product formation in the CSH/ML-grown culture. Thus, the kinetic models for biomass concentration, glucose consumption, and product formation were adjusted for the CSH/ML-grown culture, in which the estimated parameters are presented in Table 6. The alignment of experimental results and the model equations is shown in Figure 2. Accordingly, the correlation coefficient value was higher than 0.98, indicating that the mathematical model fitted well with the experimental data. Similar to the culture grown in NaNO_3_-defined medium, the kinetic parameters of the CSH/ML-grown culture showed the phenomena of the growth-associated products of DGLA and lipid. However, the values of biomass yield and maximum biomass titer in the CSH/ML-grown culture were lower than those grown in the NaNO_3_-defined medium. In addition, the specific product turnover rate (*k*_*dD*_) and degree of product depletion (*n*_*D*_) in the CSH/ML-grown culture after the exhaustion of carbon substrate were higher than the cultures grown in NaNO_3_-defined medium, which are in agreement with the sharp decrease in the DGLA and lipid titers. Notably, the maximum lipid titer (*L*_*max*_) of the CSH/ML-grown culture (1.22 g lipid/L), which was computed from the model, was lower than that of the NaNO_3_-defined medium (1.84 g lipid/L) as shown in Table 7.

**TABLE 7:** Comparison between the kinetic parameters of the generated model of the DGLA-producing strain of *A. oryzae* and those of other oleaginous yeasts and fungi (data modified from Diamantopoulou et al., 2020).

Strains	Parameters						References
	*X*_*m*_ (g/L)	*μ_*max*_* (h^–1^)	*Y_*X*__/__*S*_* (g*_*X*_*/g*_*S*_*)	*L*_*max*_ (g/L)	β*_*L*_* (g/g.h)	DGLA (mg/L)	
DGLA-producing strain							
NaNO_3_-defined medium	18.39	0.16	0.34	1.84	6.06 × 10^–8^	164.83	This study^*a*^
CSH/ML medium	13.72	0.17	0.21	1.22	1.49 × 10^–8^	170.43	This study^*a*^
*Mucor circinelloides*	ND	0.13	0.49–0.51	ND	0.06	ND	Aggelis and Sourdis, 1997^*b*^
*Mortierella isabellina*	ND	0.47	0.35	ND	ND	ND	Economou et al., 2011^*b*^
*Cryptococcus curvatus*	14.60–18.70	0.052–0.093	0.33–0.54	3.60–11.70	3.00 × 10^–3^	ND	Diamantopoulou et al., 2020^*b*^
*Yarrowia lipolytica*	ND	0.23	0.78	2.70	0.10	ND	Papanikolaou and Aggelis, 2003^*b*^
*Rhodotorula glutinis*	ND	0.27	0.20	ND	0.05	ND	Karamerou et al., 2016^*b*^
*Apiotrichum curvatum*	ND	0.19	0.69	ND	0.025	ND	Glatz et al., 1984^*b*^

**^*a*^The kinetic parameters of cell growth and lipid-containing biomass of the engineered strain of A. oryzae in this study. ^*b*^The kinetic parameter of cell growth and lipid-free biomass of other oleaginous strains. ND stands for no data.**

## Discussion

The economically viable process for DGLA production is of much attention due to a prospect in its applications in several industrial segments. In addition to the cell mass production, two consolidated metabolic processes, including PUFA biosynthesis and lipid-accumulating process, contribute to the DGLA production yield. Although the *n*-6 PUFA biosynthetic pathway in the oleaginous *A. oryzae* was genetically engineered (Chutrakul et al., 2016), the production of DGLA-enriched lipid is somewhat unexplored. In this work, the physiological manipulation in the fungal growth and lipid production aiming to establish the production process for DGLA-enriched lipid was implemented in addition to the genetic modification. Two main parameters involved in the growth performances, including nutrient source and fermentation condition, were assessed in association with its lipid phenotypes. Unexpectedly, the biomass concentration of the engineered strain using yeast extract was less than the cultures using nitrate sources, which is in contrast with the published research works, mentioning that yeast extract was the best nitrogen source for fermentation of several filamentous fungi, such as *Aspergillus* sp. and *Fusarium oxysporum* (Colla et al., 2016; Matsakas et al., 2017). Actually, the nitrogen source suitable for cell growth might not enhance lipid production. However, we found that NaNO_3_ was the best nitrogen source for biomass and lipid productions of the engineered strain of *A. oryzae*. It has been previously reported that nitrate was a favored nitrogen source for lipid production of *Aspergillus versicolor* (Karatay and Dönmez, 2014). The DGLA-producing strain of *A. oryzae* was able to grow well on a wide range of pH (3.0–6.0); however, the constant pH 6.0 was optimal for fermentation, particularly in the DGLA production that was also the same condition for maximizing the biomass production. This mild acidic condition also overcomes the cross-contamination during the fungal fermentation at larger scale. In addition to a drawback in terms of the cultivation processing, 67.2–73.6% decrease in lipid titer was found in the culture grown at pH 7.0, whereas the biomass decreased about 17.4–36.4% as compared with those cultivated at other pH conditions. Possibly, the fungal cell might channel the carbon flux to other metabolic pathways rather than the lipid biosynthesis, and had more energy expenditure for cell acclimatization to such neutral pH environment.

As the results of the cultivations with nitrogen and pH variables, the concomitant increases in DGLA and lipid titers along the fungal growth (biomass production) referring their linear correlations indicated that both 20C*-*PUFA and lipid were growth-associated metabolites of the engineered *A. oryzae* strain. These results are also supported by the mathematical models with the β*_*D*_* and β*_*L*_* values close to zero that were generated for describing behaviors of the fungal cultures using NaNO_3_-defined and CSH/ML media. These might be explained by the structure role of PUFAs as constituents in biological membranes. It has been previously documented that DGLA and GLA were mostly esterified into phospholipid fraction of this engineered strain of *A. oryzae* even though they were also distributed into the neutral lipids, particularly in triacylglycerol (Chutrakul et al., 2016). However, it has been previously reported that the lipid over-production in oleaginous species is usually found at the end of growth phase, called lipid-accumulating phase, in which the nitrogen source is depleted and carbon source remains available (Papanikolaou and Aggelis, 2011; Lamers et al., 2016). In our work, the carbon-limited condition was elaborated at the lipid-accumulating phase of the engineered strain, although such condition could not boost the lipid content over 20% of dry biomass, as known for the phenotype of oleaginous strains (Ochsenreither et al., 2016; Gientka et al., 2017). Generally, the cultivation at high C/N ratio has been supposed to be a strategy for enhancing the lipid content in biomass (Lopes et al., 2020), but the PUFAs proportionally decreased in total lipid, pertaining a poor quality of PUFA-containing oils. For the high C/N ratio condition, the residual glucose usually remains in the biowaste derived from the production process in addition to the drawback in high medium cost that might be attributed to the overall operation cost for PUFA production.

When using the cheap CSH medium, the biomass titer was low, which corresponded to the high *m*_*S*_ value, thus leading to low DGLA titer as compared with the culture grown in NaNO_3_-defined medium. Although both media had the same C/N ratio of 30, it seems likely that the fungal cell might manipulate its metabolic energy in nutrient assimilation for the biomass production. Interestingly, the supplementation of ML into the cheap CSH medium could significantly enhance the biomass production. In addition, the significant increase in DGLA production by adding low amount of ML (1% *v*/*v*) into the CSH culture was a result of the increase of DGLA proportion up to 22.1 ± 2.3% in TFA that was very high as compared with the previous report of the same fungal strain (1.8–2.0% DGLA in TFA) (Chutrakul et al., 2016). Low DGLA proportions have also been reported for the recombinant strains of *Mucor circinelloides* (5.7% DGLA in TFA) and *Saccharomyces cerevisiae* (3.5% DGLA in TFA) even though they were challenged by cultivating at low temperatures (Yazawa et al., 2007; Khan et al., 2019). Actually, low-temperature stress has been used for enhancing the PUFA proportion, which has been thought to be involved in adaptation mechanism of the microbial cells for increasing membrane fluidity (Vigh et al., 2005; Cheawchanlertfa et al., 2011). The high DGLA proportion found in the CSH/ML culture grown at normal temperature can be possibly explained by the effect of predominant amino acids (glutamic acid and aspartic acid) in ML. This result coincided with the previous report that glutamate supplementation promoted the growth of *Mortierella* culture by accelerating substrate metabolism, and thus arachidonic acid production was enhanced (Yu et al., 2003). Moreover, it has been proposed that the aspartate and glycine metabolisms might involve in leveraging between biomass and lipid production as their altered metabolic fluxes through the comparative analysis of *M. circienelloides* strain WJ11 and CBS277.49 (Isarankura Na Ayudhya et al., 2019). Similarly, GLA proportion (25.4 ± 1.3% in TFA) in the engineered *A. oryzae* culture grown at the optimal CSH medium was higher than that found in the same fungal strain (9.5–16.0% GLA in TFA) (Chutrakul et al., 2016) and the known GLA-producing strains of Zygomycete fungi, such as *Mucor rouxii* (18.1% GLA in TFA), *Cunninghamella* sp. (11.9% GLA in TFA), and *Mortierella alpina* (1.6–6.1% GLA in TFA) (Kavadia et al., 2001; Jeennor et al., 2006; Mironov et al., 2018). Our results revealed that the change in fatty acid phenotypes might be attained by cell acclimatization in the optimal medium apart from the influence of genetic manipulation. Moreover, it has been reported that the conversion of GLA to DGLA by Δ^6^-elongase activity was a diminished step for *n*-6 LC-PUFA biosynthesis as previously described (Iskandarov et al., 2011; Khan et al., 2019). Likely, the fungal cells preferred the accumulation of GLA over than the triene 20-carbon LC-PUFAs that might be a cellular process for maintaining membrane structure and function.

Considering the kinetic models generated from the batch fermentation of the engineered *A. oryzae* strain, the cell growth was directly proportional to the glucose consumption rate, and the lipid production was also linearly dependent to the biomass concentration (Table 6). Accordingly, the glucose was considered to be the limiting substrate for cell growth and lipid production. It has been recently reported that the lipid production rate of *M. circinelloides* was induced by increasing the glucose uptake rate (Isarankura Na Ayudhya et al., 2019). However, it could not be clearly distinguished that lipids are primary or secondary metabolites in oleaginous strains because the fatty acids and other precursors for lipid biosynthesis could be synthesized via central metabolic pathway through glycolysis (glucose is a starting carbon precursor) and other metabolic routes, such as amino acid and lipid metabolisms at particular conditions (Vorapreeda et al., 2012; Vongsangnak et al., 2013). However, the lipid content of the engineered *A. oryzae* cultures grown in defined and CSH media decreased after glucose depletion, which is the so-called lipid turnover stage, similar to the previous reports (Aggelis and Sourdis, 1997; Papanikolaou and Aggelis, 2003). Possibly, the *A. oryzae* cells might utilize a portion of storage lipid in the cell via β-oxidation to generate energy for either cell maintenance or lipid-free biomass formation as previously described (Aggelis and Sourdis, 1997; Maggio-Hall and Keller, 2004; Papanikolaou et al., 2006; Rossi et al., 2011). In addition, it has been documented that the lipid turnover occurred particularly when some essential nutrients remained in sufficient amounts in the fermentation medium (Papanikolaou et al., 2004; Economou et al., 2010). With the exception of maximum specific growth rate, the altered kinetic values (i.e., *m*_*S*_, *k*_*dD*_, and *k*_*dL*_) found in the engineered *A. oryzae* strain when grown in different culture media (NaNO_3_-defined and CSH/ML media) indicated that the lipid metabolites are dynamic molecules in leveraging several cellular metabolisms during the cell growth, probably for maintaining cell homeostasis. It can be documented that the generated logistic equation could explained well the fungal growth, which was similar to the previous studies of other oleaginous fungi and yeast (Amirsadeghi et al., 2015; Fang et al., 2018; Xu et al., 2018). In addition, the Luedeking–Piret equation, which was modified by including the term of lipid degradation, could describe the phenomena of lipid production and degradation as well as DGLA production. When considering the kinetic models of oleaginous yeasts and fungi (Table 7), it can be presumably documented that the maximum biomass concentration and lipid production were attributed by the medium composition and cultivation condition in addition to the strain dependence. Although the growth phenotypes (i.e., specific growth rate, biomass yield on substrate, and biomass concentration) of the engineered strain of *A. oryzae* were comparable with some oleaginous strains (Karamerou and Webb, 2019; Diamantopoulou et al., 2020), it had lower lipid titer than the others. It seems likely that the fungal cell might manipulate either biosynthesis or accumulation of PUFA-rich lipid (55.3–66.7% in TFA, Tables 3, 5) under such carbon-limited condition.

Taken together, the efficient fermentation process for DGLA production of the engineered strain of *A. oryzae* was developed by using CSH/ML medium and normal temperature condition. However, we suggest that the DGLA production can be more improved by enhancing biomass titer using fed-batch fermentation mode under such carbon-limited condition for maintaining the oil constituent rich in DGLA. Noteworthy, the observation that there was a significant decrease of DGLA and lipid titers of the CSH/ML culture after the glucose exhaustion corresponding to the parameter values (*k*_*dD*_, *k*_*dL*_, *n*_*D*_, and *n*_*L*_) is meaningful in the operational practice for defining the end-point of the fermentation to obtain the maximum DGLA titer. The preliminary evaluation of the medium cost exhibited that the CSH/ML medium was cheaper than the NaNO_3_-defined medium, which accounted for 72%. However, the operating cost and other expenditures in both upstream and downstream processing for DGLA production should be further assessed to achieve the economically feasible process for industrial applications.

## Data Availability Statement

All datasets generated for this study are included in the article/ Supplementary Material.

## Author Contributions

SA performed experimental design, fermentation, article writing, and figure and table arrangement. JA carried out fungal cultivation and fatty acid and lipid analysis. SW simulated the kinetic models and analyzed the data. BK was involved in calculating the fermentation kinetic parameters and metabolite analysis. KL carried out research design, result interpretation, execution, article revision, and completion of the final article. All authors contributed to the article and approved the submitted version.

## Conflict of Interest

The authors declare that the research was conducted in the absence of any commercial or financial relationships that could be construed as a potential conflict of interest.

## Footnotes

1 http://www.berkeleymadonna.com
