# Supplementary material for: Physiological Traits of Dihomo-γ-Linolenic Acid Production of the Engineered Aspergillus oryzae by Comparing Mathematical Models
Source: Front Microbiol. 2020 Nov 5;11:546230. doi: 10.3389/fmicb.2020.546230 (PMC7674286; doi:10.3389/fmicb.2020.546230)
Supplement: Supplementary file 1 [file Data_Sheet_1.PDF]

Physiological traits of dihomo-γ-linolenic acid production of the engineered *Aspergillus oryzae* by comparing mathematic models

Sompot Antimanon, Jutamas Anantayanon, Siwaporn Wannawilai, Bhimabol Khongto and Kobkul Laoteng*

Functional Ingredients and Food Innovation Research Group, National Center for Genetic Engineering and Biotechnology (BIOTEC), National Science and Technology Development Agency (NSTDA), Thailand Science Park, Pathum Thani 12120, Thailand

* Correspondence: Kobkul Laoteng

Tel: +66-2-5646700 Fax: +66-2-5646707

kobkul@biotec.or.th

Supplementary Table S1 Fatty acid composition in TFA of the engineered strain of *A. oryzae* cultivated in different nitrogen sources.

**Nitrogen source**	**C16:0**	**C18:0**	**C18:1 *n*-9**	**C18:2 *n*-6**	**C18:3 *n*-6**	**C20:3 *n*-6**	**Others**
Yeast extract	28.5±1.9	8.1±0.2	19.0±1.4	21.6±1.4	14.6±1.1	7.8±0.4	0.4±0.0
KNO_3	21.3±2.1	7.3±1.1	22.0±1.2	24.9±1.8	15.3±1.3	9.1±0.3	0.0±0.0
$NaNO_3$	20.1±1.9	7.2±1.0	20.4±1.2	27.6±1.9	15.3±1.1	8.9±1.0	0.4±0.0
$(NH_4)_2HPO_4$	20.0±2.4	8.4±0.4	25.0±2.7	21.8±1.7	18.0±1.7	6.4±0.6	0.4±0.0
NH_4Cl	18.0±1.1	8.0±0.5	28.3±1.8	20.9±0.9	18.7±1.4	5.9±0.4	0.1±0.0
$(NH_4)_2SO_4$	19.1±1.8	8.9±0.3	28.5±2.8	19.3±0.9	17.2±0.8	6.8±1.1	0.3±0.0

All data are presented as mean values with standard deviation (SD).

Supplementary Table S2 Amino acid profiles and other nutrients of ML derived from the monosodium glutamate production process.

Composition	Amount	Unit
**Amino acids**		
Alanine	11.99	mg/g
Arginine	nd	mg/g
Aspartic acid	6.84	mg/g
Cysteine	nd	mg/g
Glutamic acid	60.71	mg/g
Glycine	1.04	mg/g
Histidine	nd	mg/g
Isoleucine	<1.00	mg/g
leucine	1.04	mg/g
lysine	1.20	mg/g
Methionine	nd	mg/g
phenylalanine	nd	mg/g
Proline	nd	mg/g
Serine	nd	mg/g
Threonine	nd	mg/g
Tyrosine	nd	mg/g
Tryptophan	nd	mg/g
Valine	2.12	mg/g
**Protein**	0.35	mg/g
**Total N**	6.35	% (w/v)

"nd" indicates the undetectable amino acids
